# The Impact of Health Information Exposure and Source Credibility on COVID-19 Vaccination Intention in Germany

**DOI:** 10.3390/ijerph18094678

**Published:** 2021-04-28

**Authors:** Volker Gehrau, Sam Fujarski, Hannah Lorenz, Carla Schieb, Bernd Blöbaum

**Affiliations:** Department of Communication, University of Muenster, 48149 Münster, Germany; s.fujarski@uni-muenster.de (S.F.); hannah.lorenz@uni-muenster.de (H.L.); carla.schieb@uni-muenster.de (C.S.); bloebaum@uni-muenster.de (B.B.)

**Keywords:** COVID-19, health information sources, vaccination intention, exposure to health information sources, source credibility, Germany

## Abstract

Due to the novelty and high transmission rate of the coronavirus disease 2019 (COVID-19), direct medical countermeasures are urgently needed. Among actions against the further outbreak of COVID-19, vaccination has been considered as a chief candidate. However, the rapid development of COVID-19 vaccines has led to concern about their safety and thus to public vaccine hesitancy. Strategic heath communication channels, which are widely used and highly trusted, can contribute to more effective promotions of vaccination intention and to the reduction of misleading information about COVID-19 vaccines. Therefore, this study examines the relationship between the exposure to and credibility of different health information sources and the COVID-19 vaccination intention among 629 German adults. Descriptive statistical analysis and multiple linear regressions are employed to examine the research questions. Results reveal that, aside from reliable information from experts and health authorities, local newspapers also have a positive impact on COVID-19 vaccination intention. However, this effect diminishes to some extent when age is considered. In addition, alternative information sources pose a noticeable threat to COVID-19 vaccination intention. Therefore, a close cooperation between healthcare experts, health authorities, and mass media with regard to information dissemination is conducive for vaccination campaigns and for the fight against misleading claims about COVID-19 vaccines.

## 1. Introduction

Vaccine hesitancy is one of the biggest threats in a health pandemic, potentially causing severe long-term consequences [1]. Because of the high transmission rate of the coronavirus without specific treatment, scientists are currently working on the development of COVID-19 vaccines. By the time of the survey, more than 200 vaccines had been developed, yet only few were proven to be efficient and safe for use [2,3]. At this time in Germany, the number of new COVID-19 infections per 100,000 inhabitants over a seven-day-period (7-day incidence) amounted to approximately 140 cases [4]. For the examined region, the reported 7-day incidence was 94 cases [4]. In Germany, the 50-case-threshold of the 7-day incidence was set to be critical and protective measures and restrictions were to be taken immediately [5]. However, by the time of the survey, no vaccine had received regulatory approval for Germany and the first vaccination campaigns were still in preparatory stage. As of April 2021, three vaccines have been approved in Germany, including BioNTech/Pfizer (since 26 December 2020), Moderna (since 14 January 2021), and AstraZeneca (since 8 February 2021) [6]. The rapid development of the COVID-19 vaccines has led to mistrust and concern about the safety of the vaccines, which directly increases public vaccine hesitancy [7]. Numerous misleading, unproven claims against COVID-19 vaccines were widely spread through social media, thereby negatively influencing vaccination intention [8]. Strategic health communication is considered an important factor in raising public awareness about immunization and increasing general knowledge about vaccination. Systematic literature reviews on vaccines and vaccination indicate that exposure to health information through different sources has both positive and negative effects on vaccination [9]. The categorization of health information sources according to the level of exposure and credibility can offer insight into the profiles of vaccine advocates and refusers, which can help us design more sophisticated vaccination programs with a more target-specific approach.

Health information orientation, understood as “the extent to which the individual is willing to look for health information” [10], is a key driver for health information seeking and exposure. Primary health information sources include online sources [11], health professionals, family and friends, mass media, and public health agencies [10,12]. Findings show gender differences [13,14] and differences regarding health orientation motives [10,15]. However, exposure to selected health information sources may differ in health crisis situations [12]. In a global health emergency like the COVID-19 pandemic, in which people deal with a novel infectious disease that was hardly known before, they may search for information from a wider range of sources to inform themselves on the ongoing pandemic [16,17]. Broadcast media (television and radio), print media (newspapers and magazines), and social media (Facebook, Instagram, Twitter, etc.) are then considered to be the most widely used health information sources [17,18]. Especially traditional media, such as broadcast and print media, were found to be the primarily used sources during infectious disease outbreaks [16,19,20,21]. Information sources such as healthcare professionals, family, and friends as well as public health organizations were also perceived to be important health information sources in health crisis contexts [12]. A significant part of the population turns towards alternative news media and uses them as a source of health information as well. These types of information sources were often “suspected of spreading rumors and misleading information opposing the view of traditional news media and further contributing to the insecurity and confusion among the population” [22]. Therefore, health information sources may influence individuals’ attitude on vaccination and consequently their willingness to be vaccinated [17,19,23,24,25,26,27]. There still exists, however, little knowledge concerning the impact of exposure to health information sources on COVID-19 vaccination intention or hesitancy. Knowledge of the relationship between exposure to health information sources and vaccination intention may help us combat misinformation and organize vaccination campaigns effectively.

Public compliance with a government’s recommended health prevention measures such as vaccination plays a vital role in vaccination campaigns as well as in controlling and reducing the impact of a health pandemic [28,29]. Trust in health information providers, which is based on the credibility of their information, was found to be a significant antecedent of compliance [29]. According to recent studies, healthcare professionals are consistently regarded as the most highly trusted sources of health information [16,30,31,32,33,34]. Yet, although they are often perceived as trustworthy sources of information, they are not always the first source used for health information [33,35]. This finding could be explained by limited access to this kind of information source. For example, long waiting times and a perceived lack of treatment and counseling time on behalf of physicians may discourage patients from asking additional questions or seeking detailed explanations [12,35]. Interpersonal information sources such as family and friends or institutional information sources such as government information sources or health authorities are also considered trustworthy in health-related issues [27,32,33]. These types of information sources become even more important in health crisis situations [29]. Compared to the aforementioned health information sources, mass media and social networks have the lowest degree of trust [27,29,33]. However, the individual level of perceived trust in health information sources is not always proportional to the exposure to those sources. Despite their relatively low credibility levels, traditional media were the most commonly cited sources during crisis situations [12,16,19,27]. In particular, the Internet, due to its unconstrained access, is reported to be the “source of first resort” for general health issues [30,33]. Mass media were seen as “critical in making them [participants] aware of the crisis” [19]. Promoting vaccines through channels which are both highly trusted and widely used can greatly contribute to the reduction of confusion caused by misleading information about vaccination and therefore to the success of vaccination campaigns.

For this reason, our current study aims to disclose the relationship between exposure to and the credibility of different health information sources as well as the age-related discrepancy regarding vaccination intention among German adults. Therefore, we examined the following research questions:

RQ1: Which health information sources were mostly used by the German population in times of COVID-19?
RQ2: Which health information sources were most trusted by the German population in times of COVID-19?
RQ3: Does exposure to health information sources have an effect on COVID-19 vaccination intention?
RQ4: Does the credibility of health information sources have an effect on COVID-19 vaccination intention?
RQ5: How do exposure to and confidence in information sources interact in their impact on COVID-19 vaccination intention?

## 2. Materials and Methods

Data collection took place from 23 November to 7 December 2020. We limited the survey to a largely rural region in western Germany that encompasses one large city of approximately 300,000 inhabitants. The study was funded by the German Federal Ministry for Education and Research (BMBF) as part of the ELSA program, which explores ethical, legal, and social aspects of modern life sciences in Germany. The aim of the study was to investigate exposure to and credibility of various sources of health information, changes with regard to individual health behavior, and the vaccination intention of German adults during the second COVID-19 wave in November 2020. We used random-quota sampling methods to compile a sample representing the entire population in terms of the following demographic categories: age, sex, and education level. Therefore, a combination of online and telephone survey was applied, so that all age groups between 18 and 80 years could be covered representatively. The study was administered by a professional German opinion research institute.

### 2.1. Sample

The data were collected from a sample of 629 respondents, 509 of whom completed an online survey and 120 a telephone survey. The sample is broadly representative of a region in western Germany. The sample includes 53 percent women and 47 percent men, with 19 percent representing a low educational level, 29 a medium level, and 51 percent representing a high educational level. Respondents had an average age of 51 (*M* = 51.3, *SD* = 17.0).

### 2.2. Survey and Measures

The conducted survey included various aspects of health information behavior and health behavior in general. In the first block of questions we focused on 13 different information sources that respondents used to obtain health information during the second COVID-19 outbreak. Respondents were asked “How often did you use the following sources to get information about health-related issues?” These questions comprised response options on a 5-point scale from 0 (not at all) to 4 (daily). The second set of questions addressed perceived trust levels for the aforementioned information sources. The respondents rated trust on a 4-point scale from 1 (strongly distrust) to 4 (strongly trust). These two sets of questions were based on a battery of measures from the German Health Information National Trends Surveys (HINTS Germany), which employs systematic and continuous monitoring of health information behavior among the German population [14]. Finally, COVID-19 vaccination intention was measured with a single item: “How likely or unlikely are you to get vaccinated against the COVID-19 virus if an approved vaccine became available?”. Possible answers were: very unlikely, fairly unlikely, neither unlikely nor likely, fairly likely, and very likely, which were scored from 0 to 4. In addition, a score was created to examine the effect of source credibility (trust) in combination with health information exposure (usage). For this purpose, both values, usage and trust, for each examined information source were multiplied. In case the trust score did not apply because a certain source was not used, the score was coded as 0. If no information was available for usage or if the source was used but no information was available for trust, the score was considered as a missing value.

## 3. Results

### 3.1. Descriptive Statistics of Usage and Trust

First, interest is directed toward the use of sources to obtain health-related information. Table 1 provides a summary of the usage of different health information sources. Television (*M* = 2.99, *SD* = 1.27) was the most frequently used source. Radio (*M* = 2.58, *SD* = 1.44) and local newspapers (*M* = 2.43, *SD* = 1.53) followed at some distance. They were succeeded by family (*M* = 2.06, *SD* = 1.42) and public health authorities (*M* = 1.90, *SD* = 1.43). Internet sources (*M* = 1.76, *SD* = 1.53), national newspapers (*M* = 1.50, *SD* = 1.42), social networks (*M* = 1.38, *SD* = 1.05), and alternative media sources (*M* = 1.21, *SD* = 1.21) were used less frequently as sources of health information. Medical professionals (*M* = 1.10, *SD* = 1.43) and scholarly sources such as scientists (*M* = 1.02, *SD* = 1.28) were the least used sources.

During the COVID-19 pandemic, traditional mass media featured as the primary source from which people most frequently retrieved information about health issues, whereas social networks or alternative media—common sources of false claims or conspiracy theories about COVID-19—were rarely used in health information seeking. This raises the question of how much trust is placed in the aforementioned sources. Table 2 displays an overview of the trust levels for each examined health information source.

Medical professionals (*M* = 3.29, *SD* = 0.68), health authorities (*M* = 3.27, *SD* = 0.65), or scientists (*M* = 3.18, *SD* = 0.68) were trusted most, followed by family (*M* = 2.88, *SD* = 0.75) and then legacy media: radio (*M* = 2.83, *SD* = 0.68), local newspapers (*M* = 2.82, *SD* = 0.68), television (*M* = 2.65, *SD* = 0.82), and national newspapers (*M* = 2.65, *SD* = 0.62). At the very end of the credibility list, one finds the Internet (*M* = 2.20, *SD* = 0.69), alternative sources of information (*M* = 1.86, *SD* = 0.70), and social networks (*M* = 1.64, *SD* = 0.72). In addition to the reversed order, the level of homogeneity within the data was striking. Although the scales were identical, the standard deviations in the estimation of trust were only roughly half as large as those in the estimation of usage.

### 3.2. Interaction between Usage and Trust

**Figure 1:**
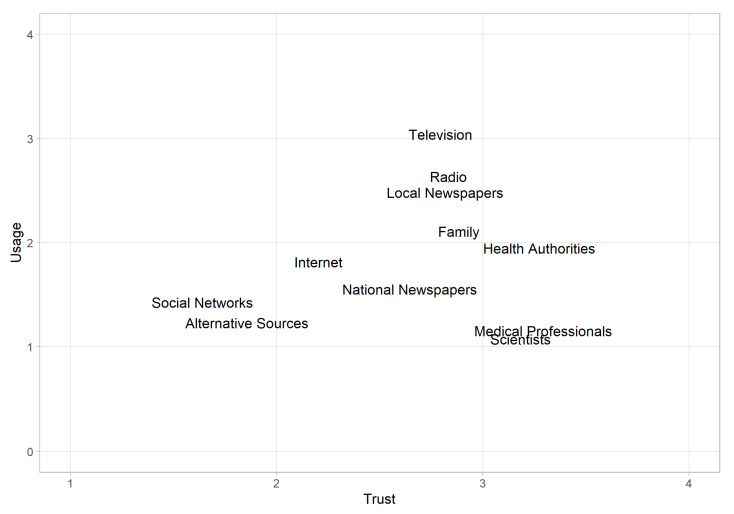
Scatterplot for the interaction between usage and trust.

When making strategic decisions about which communication channels to use to provide information about vaccination and to increase vaccination intention, both aspects, usage and trust, should be considered simultaneously, since it is likely that only sources that are widely used *and* highly trusted can be more effective. To verify this assumption, the mean values of usage and trust for each information source were displayed in a scatterplot (Figure 1). The *Y*-axis represented usage and the *X*-axis represented trust. Information sources in the top right quadrant with higher usage and greater trust levels were considered relevant and useful channels for disseminating health information.

This assumption reflects the difficulty of planning vaccination campaigns. High trust scores in medical professionals and in information stemming from scientists is of little use, since information from these sources is not commonly used. Information from health authorities is more suitable, because not only are they highly trusted sources, they are also used on a more regular basis. Traditional mass media, however, are probably the best way to disseminate information on vaccination, because they are used frequently and are assigned at least a medium degree of trust. Therefore, we examined the interaction of both trust and usage resulting from their product.

The product would be zero, regardless of trust, if a source was not used at all. The product tended to be low when a source was rarely used and barely trusted, and it was high when both usage and trust reached high scores. Table 3 displays an overview of the interaction between usage and trust for each examined information source. According to this analysis, television was clearly in the lead (*M* = 8.59, *SD* = 4.44), followed by radio (*M* = 7.62, *SD* = 4.72), and the local newspapers (*M* = 7.27, *SD* = 5.15). Public health authorities (*M* = 6.45, *SD* = 4.44) and family (*M* = 6.16, *SD* = 3.55) scored well as information sources too. National newspapers (*M* = 4.16, *SD* =3.56), the Internet (*M* = 4.03, *SD* = 3.72), medical professionals (*M* = 3.68, *SD* = 3.74), and scientists (*M* = 3.42, *SD* = 4.27) reached average numbers. Social networks (*M* = 2.71, *SD* = 5.18) and alternative sources of information (*M* = 2.59, *SD* = 4.41) ranked at the bottom of the list. High standard deviation was striking for all values. The interaction of usage and trust, thus, varied greatly from individual to individual. This was the case especially for local newspapers and social networks. According to this finding, traditional mass media including television, radio, and local newspapers in combination with information from public health authorities would be the best way to promote COVID-19 vaccines and increase vaccination intention.

### 3.3. Effect of Usage and Trust on COVID-19 Vaccination Intention

Finally, three linear multiple regression analyses were conducted to predict vaccination intention (Table 4). In the first regression analysis, usage of each information source was used as predictor, the second regression analysis applied trust as a predictor, and the last regression analysis made use of the interaction between usage and trust as a predictor.

The first regression model was highly significant (*F* (11,557) = 5.96, *p* < 0.001), explaining, however, a mere nine percent of the variance of vaccination intention (adj. *R*^2^ = 0.09). Four predictors had a significant influence on vaccination intention (Table 4). However, the effect of the two strongest predictors (social networks and alternative sources) were negative: the more these sources were used, the lower the willingness to get vaccinated. This was especially true for the usage of alternative information sources, having a standardized regression coefficient of *β* = –0.17, followed by social networks with *β* = –0.16. This dynamic was thwarted by the positive effects of local newspapers with a coefficient of *β* = 0.14 and information from scientists with *β* = 0.12. Respondents who turned to these two sources fairly often were more likely to vaccinate.

The second regression model using trust variables as predictors scored significantly higher. It explained 24 percent of the variance of vaccination intention (adj. *R*^2^ = 0.24) and was highly significant (*F*(11,450) = 14.01, *p* < 0.001). In this model, most predictors had a positive effect. The greater the trust placed in health information sources, the higher the vaccination intention. Trust in television was the strongest predictor for vaccination intention with *β* = 0.26. Trust in health authorities (*β* = 0.14), in local newspapers (*β* = 0.12), and in scientists (*β* = 0.12) also showed a positive effect on vaccination intention. In contrast, a negative effect of trust in alternative information sources (*β* = −0.16) on vaccination intention was found. Individuals trusting alternative information sources were less likely to vaccinate.

The third regression model with the interaction of usage and trust as predictors showed a medium goodness of fit, with 14 percent explained variance of vaccination intention (*F*(11,515) = 8.76, *p* < 0.001, adj. *R*^2^ = 0.14). In this model, positive and negative effects were almost equal. Television (*β* = 0.21), local newspapers (*β* = 0.15), and scientists (*β* = 0.10) showed a positive effect on vaccination intention. On the other hand, alternative sources (*β* = −0.19), social networks (*β* = −0.10), and family (*β* = −0.10) had a negative effect.

The following findings are noteworthy as we compared three different models (Table 4). In all three models, the strongest consistent effect came from alternative sources of information. Those using or trusting this information source on a higher level were less likely to vaccinate. In comparison to alternative information sources, the effects of local newspapers and scientists were slightly weaker. The greater extent of usage and trust in these sources led to a stronger vaccination intention. In two regression models (with trust and usage × trust as predictors) television turned out to be the strongest predictor for vaccination intention. Hence, it became obvious that the frequency of television usage alone did not play a substantial role in vaccination intention. In this case, the usage of social networks was found to be more important for vaccination intention. A significant negative effect (*β* = −0.09) of family as an information source was only found for the regression model that calculated the interaction between usage and trust. However, this effect was small and should not be considered relevant.

Comprehensive representative studies on media use in Germany suggest significant age differences [36,37,38]. In particular, the usage of online social networks is significantly more widespread among younger than among older people [39]. For newspaper usage, traditionally it is the other way around. Elderly people tend to be more willing to be vaccinated and prefer local newspapers. In consequence, it is problematic to differentiate the effects of age and usage on vaccination intention. Since the risk of severe disease progression for COVID-19 increases with age, it is also reasonable to assume an increase in vaccination intention with age. To investigate this assumption, age was included as an additional predictor in the previously examined regression models. As expected, we found a positive relationship between age and vaccination intention in all models (Table 5): vaccination intention was found to be higher among older respondents. Because of the significant effect of age, the explained variance grew significantly in all three models. The increase ranged between four and six percent. The positive effect of trust in television as well as the negative effect of alternative sources of information remained consistent. The positive effect of scientists as an information source also remained almost identical.

Adding age as a predictor had an effect on other variables as well. For example, the effect of local newspapers decreased slightly across all three models and was no longer significant when age was added. Thus, the positive effect of local newspaper usage that was detected initially (Table 4) was largely due to the effect of age on vaccination intention as well as on newspaper use. However, positive effects of local newspaper consumption still tended to be found in all models despite the age variable (Table 5). This result indicates that local newspapers could have a potentially positive effect on vaccination intention. However, this effect might not be differentiated well enough methodologically from the effect of age and should not be identified as an independent effect. A similar result was also found for social networks. The two usage effects detected in the first regression models (Table 4) were significantly lower and no longer significant when age was included (Table 5). In the new regression models with age as an additional predictor, aside from trust and the interaction of usage and trust, the usage of public health authorities alone was also found to have a positive effect on vaccination intention (*β* = 0.10).

## 4. Discussion

The current study revealed some important insights for optimizing strategic communication in order to increase COVID-19 vaccination intention. On the one hand, mass media like television, radio, and newspapers were the sources Germans used most often to obtain information about health issues in contrast to experts, who seldomly feature as information sources. On the other hand, people showed more trust in health-related information from experts than information from mass media. Taking the interaction of trust and usage into account, health authorities seem most appropriate to spread information about COVID-19 vaccination, since information from health authorities is both widely used and largely trusted. Mass media can also be considered an appropriate channel for disseminating health-related knowledge, since they are used often and are ascribed a sufficient level of trust. In contrast, information from social networks or alternative information sources can claim neither sufficient usage nor sufficient trust levels, and are thus hardly useful for increasing vaccination intention.

From this point of view, strategic health communication should provide information stemming from experts and distributed via the mass media as well as official health authorities. However, this approach might not be the best way to communicate about COVID-19 vaccination. Communication about COVID-19 vaccination could vary since vaccination intention and the usage of mass media differ strongly by age group: older people are more willing to accept a COVID-19 vaccine and use newspapers more often than younger people. In contrast, younger people show a lower COVID-19 vaccination intention and use Internet sources and social networks more often than older respondents.

Information coming from experts and official authorities has a positive impact on COVID-19 vaccination intention. Those using information originating from these sources and placing a high level of trust in them are more inclined to get vaccinated. In addition, we detected a strong positive effect with regard to trust in health-related television content. Those who have more trust in television-based health information are more willing to vaccinate. Furthermore, the usage of and trust in local newspapers’ health reporting have a positive effect on COVID-19 vaccination intention, which partly diminishes, however, when age is considered. Nevertheless, even after controlling for age, the strongest non-significant positive effect appears between newspapers and COVID-19 vaccination intention. One interpretation could be that the effect of age on vaccination intention has the strongest impact but cannot be separated from the effect generated by newspaper consumption. Another reason might be that usage of and trust in newspapers enhances vaccination intention especially among the elderly.

The most surprising effect surfaced when taking a close look at alternative information channels. This type of source is used only seldomly to obtain information about health-related issues, and most people place very little trust in information from alternative sources. Nevertheless, the usage of and trust in these sources systematically lower COVID-19 vaccination intention. This is a highly relevant conclusion when it comes to planning COVID-19 vaccination campaigns. Even though fake news and misleading information disseminated by alternative information channels may reach a small public only and are considered unreliable by a large majority, this type of source might still be an important factor working against COVID-19 vaccination intention. The small group of people who have been negatively influenced by alternative information sources are likely to distrust COVID-19 vaccination campaigns, which may somehow affect others. Therefore, it seems necessary to counter such misinformation. A promising approach could be to grant health experts airtime in mass media, especially on television.

### 4.1. Limitations

The study presented in this paper comprises two major limitations. Firstly, the sample might be considered a weakness because it is limited to one region only, which is not representative of Germany as a whole. On the other hand, the region examined by this study is not atypical for larger regions in Germany. It comprises one large city with more than 300,000 inhabitants, three counties close to this city, and one county in a more rural area. In addition, the sample offers very good coverage of different age groups with a high response rate even for elderly people at ages above 70.

A more serious problem might be caused by the scales used in the study. The trust items were measured on a four-point scale and media usage was measured on a five-point scale. Both scales were adopted from other studies to assess the prevalence of information sources and the distribution of trust in society. Even though the scales are well established, they are not an optimal fit for being used as predictors in a linear regression model.

### 4.2. Future Research

To conclude, the study would like to offer some suggestions for future research. Future research should address the following problems at least. First of all, the most important influence on vaccination intention is related to high trust levels ascribed to television-based health information. In the study at hand, focus was laid merely on the extent of individual television consumption, thereby neglecting the actual content. Hence, we highly recommend conducting content analyses of the media coverage on COVID-19 vaccination. Secondly, a closer look should be taken at the effect of local newspaper usage. This might prove helpful for two reasons. The majority of studies take national newspapers into account rather than local outlets. However, our results indicate effects for local newspapers only, yet no effects for national newspapers, in the context of health information and COVID-19 vaccination. What is more, local newspaper usage correlates highly with age, another reason for future research employing this emphasis. In order to analyze in detail the effect of local newspaper consumption as well as ascribed trust, gathering data from larger samples seems promising. Extensive samples would allow the researcher to analyze differences in usage and trust within smaller age segments in order to evaluate whether an effect of local newspapers on vaccination intention can be confirmed after all. Finally, the negative impact of usage of and trust in alternative sources of health information should be analyzed in greater detail. As mentioned above, the effect of alternative information sources originates from a small group of people obtaining this type of information. The crucial question is how such effects can be identified by linear regression with a representative sample. One explanation might be that the usage of alternative sources is related to other variables, which in turn might be related to COVID-19 vaccination intention. Another explanation suggests that little information only might suffice to undermine COVID-19 vaccination intention.

## 5. Conclusions

For Germans, mass media and interpersonal communication are the most commonly used sources for health information. In contrast to this, experts and health authorities constitute the sources that are most trusted by Germans. Therefore, strategic health communication would best be executed in close cooperation between experts, health authorities, and mass media. The Internet and social networks, in turn, may be neglected when designing communication measures directed at all age groups. In times of the COVID-19 pandemic, health communication is crucial for informing the public about important health policies, including vaccination campaigns.

Vaccination is considered one of the most promising means for stopping the spread of COVID-19. In order for the vaccination campaign to be effective, appropriate information should be communicated by health experts and official health authorities, and disseminated via television news and local newspapers. Television still reaches a large part of the German population, and health-related information aired on television can enhance COVID-19 vaccination intention among those who consider television a trustworthy medium. In addition, the usage of local newspapers can have a positive effect on vaccination intention among the elderly. These results notwithstanding, when it comes to COVID-19 vaccines, one must bear in mind the negative impact of health-related information stemming from alternative sources, since they tend to publish misleading information and fake news. Such effects have to be considered when planning and carrying out strategic communication measures revolving around the issue of COVID-19 vaccines.

Last but not least, our results underline the importance of studying the effects of fake news and misinformation diffused by alternative information sources on different aspects of our individual and social life.

## Figures and Tables

**Table 1:** Descriptive statistics of usage of different health information sources.

Information Source	N	Min	Max	Mean	SD
Television	627	0	4	2.99	1.27
Radio	626	0	4	2.58	1.44
Local newspapers	626	0	4	2.43	1.53
Family	622	0	4	2.06	1.42
Health authorities	619	0	4	1.90	1.34
Internet	625	0	4	1.76	1.53
National newspapers	624	0	4	1.50	1.42
Social networks	620	0	4	1.38	1.05
Alternative sources	587	0	4	1.18	1.21
Medical professionals	624	0	4	1.10	1.43
Scientists	612	0	4	1.02	1.28

**Table 2:** Descriptive statistics of trust in different health information sources.

Information Source	N	Min	Max	Mean	SD
Medical professionals	608	1	4	3.29	0.68
Health authorities	607	1	4	3.27	0.65
Scientists	590	1	4	3.18	0.68
Family	576	1	4	2.88	0.75
Radio	598	1	4	2.83	0.68
Local newspapers	606	1	4	2.82	0.68
Television	613	1	4	2.79	0.82
National newspapers	550	1	4	2.65	0.62
Internet	560	1	4	2.20	0.69
Alternative sources	506	1	4	1.86	0.70
Social networks	552	1	4	1.64	0.72

**Table 3:** Descriptive statistics of the interaction between usage and trust.

Information Source	N	Min	Max	Mean	SD
Television	618	0	16	8.59	4.44
Radio	613	0	16	7.62	4.72
Local newspapers	615	0	16	7.27	5.15
Health authorities	612	0	16	6.45	4.44
Family	595	0	16	6.16	3.55
National newspapers	599	0	16	4.16	3.56
Internet	590	0	16	4.03	3.72
Medical professionals	618	0	16	3.68	3.74
Scientists	607	0	16	3.42	4.27
Social Networks	606	0	16	2.71	5.18
Alternative sources	550	0	16	2.59	4.41

**Table 4:** Linear regression models for vaccination intention.

	Indicator of Vaccination Intention		
	Usage	Trust	Usage*Trust
**Predictor**	*B*		
Television	0.08	0.26 ***	0.21 ***
Radio	−0.04	0.02	−0.02
Local newspapers	0.14 **	0.12 *	0.15 **
National newspapers	−0.03	−0.04	0.00
Internet	0.00	−0.06	0.02
Social networks	−0.16 **	0.00	−0.10
Alternative sources	−0.17 ***	−0.16 **	−0.19 ***
Medical professionals	−0.04	0.02	0.00
Scientists	0.12 *	0.12 *	0.10 *
Health authorities	0.09	0.14 *	0.08
Family	−0.05	0.00	−0.09 *
DF	577/11	461/11	515/11
F	5.96	14.01	8.76
Adj. R^2^	0.04	0.24	0.14

* *p* < 0.05; ** *p* < 0.01; *** *p* < 0.001.

**Table 5:** Linear regression models for vaccination intention with age as an additional predictor.

	Indicator of Vaccination Intention		
	Usage	Trust	Usage*Trust
**Predictor**	*β*		
Age	0.27 ***	0.26 ***	0.24 ***
Television	0.01	0.18 ***	0.14 **
Radio	0.00	0.05	0.01
Local newspapers	0.07	0.07	0.08
National newspapers	−0.03	−0.02	0.00
Internet	0.03	−0.04	0.04
Social networks	−0.06	0.03	−0.04
Alternative sources	−0.18 ***	−0.15 ***	−0.20 ***
Medical professionals	−0.05	0.02	−0.01
Scientists	0.12 **	0.10 *	0.11 **
Health authorities	0.10 *	0.19 ***	0.11 **
Family	−0.02	−0.03	−0.06
DF	556/12	449/12	514/12
F	8.47	17.11	10.53
Adj. R^2^	0.14	0.30	0.18

* *p* < 0.05; ** *p* < 0.01; *** *p* < 0.001.

## Data Availability

After the research project has been completed, the data will be made accessible at the GESIS database.

## References

[1] Schmid P., Rauber D., Betsch C., Lidolt G., Denker M.-L. (2017). Barriers of Influenza Vaccination Intention and Behavior—A Systematic Review of Influenza Vaccine Hesitancy, 2005–2016. PLoS ONE.

[2] Parker E.P.K., Shrotri M., Kampmann B. (2020). Keeping Track of the SARS-CoV-2 Vaccine Pipeline. Nat. Rev. Immunol..

[3] Polack F.P., Thomas S.J., Bailey R., Swanson K.A., Roychoudhury S., Koury K., Kalina W.V., Cooper D., Frenck J.R.W., Ping C4591001 Clinical Trial Group (2020). Safety and Efficacy of the BNT162b2 mRNA Covid-19 Vaccine. N. Engl. J. Med..

[4] Koch-Institut R. (2021). Gesamtübersicht der pro Tag ans RKI übermittelten Fälle, Todesfälle und 7-Tage-Inzidenzen nach Bundesland und Landkreis. https://www.rki.de/DE/Content/InfAZ/N/Neuartiges_Coronavirus/Daten/Fallzahlen_Daten.html;jsessionid=9E8E5096D6F9F4EADC475633EA332865.internet092?nn=13490888.

[5] Bundeskanzlerin mit den Regierungschefinnen und Regierungschefs der Länder (2020). Beschluss TOP Bekämpfung der SARS-Cov2-Pandemie. https://www.bundesregierung.de/resource/blob/975226/1798920/9448da53f1fa442c24c37abc8b0b2048/2020-10-14-beschluss-mpk-data.pdf?download=1.

[6] Koch-Institut R. (2021). Digitales Impfquotenmonitoring zur COVID-19-Impfung. https://www.rki.de/DE/Content/InfAZ/N/Neuartiges_Coronavirus/Daten/Impfquoten-Tab.html.

[7] Dror A.A., Eisenbach N., Taiber S., Morozov N.G., Mizrachi M., Zigron A., Srouji S., Sela E. (2020). Vaccine Hesitancy: The Next Challenge in the Fight against COVID-19. Eur. J. Epidemiol..

[8] Khan Y.H., Mallhi T.H., Alotaibi N.H., AlZarea A.I., Alanazi A.S., Tanveer N., Hashmi F.K. (2020). Threat of COVID-19 Vaccine Hesitancy in Pakistan: The Need for Measures to Neutralize Misleading Narratives. Am. J. Trop. Med. Hyg..

[9] Larson H.J., Jarrett C., Eckersberger E., Smith D.M.D., Paterson P. (2014). Understanding Vaccine Hesitancy around Vaccines and Vaccination from a Global Perspective: A Systematic Review of Published Literature, 2007–2012. Vaccine.

[10] Dutta-Bergman M.J. (2004). Primary Sources of Health Information: Comparisons in the Domain of Health Attitudes, Health Cognitions, and Health Behaviors. Health Commun..

[11] Jacobs W., Amuta A.O., Jeon K.C. (2017). Health Information seeking in the Digital Age: An Analysis of Health Information seeking Behavior among US Adults. Cogent Soc. Sci..

[12] Avery E. (2010). Contextual and Audience Moderators of Channel Selection and Message Reception of Public Health Information in Routine and Crisis Situations. J. Public Relat. Res..

[13] Mo P.K., Malik S.H., Coulson N.S. (2009). Gender Differences in Computer-Mediated Communication: A Systematic Literature Review of Online Health-Related Support Groups. Patient Educ. Couns..

[14] Baumann E., Czerwinski F., Reifegerste D., Neter E., Bidmon S., Terlutter R. (2017). Gender-Specific Determinants and Patterns of Online Health Information Seeking: Results from a Representative German Health Survey. J. Med. Internet Res..

[15] Steger M.F., Fitch-Martin A.R., Donnelly J., Rickard K.M. (2015). Meaning in Life and Health: Proactive Health Orientation Links Meaning in Life to Health Variables Among American Undergraduates. J. Happiness Stud..

[16] Ali S.H., Foreman J., Tozan Y., Capasso A., Jones A.M., DiClemente R.J. (2020). Trends and Predictors of COVID-19 Information Sources and Their Relationship with Knowledge and Beliefs Related to the Pandemic: Nationwide Cross-Sectional Study. JMIR Public Health Surveill..

[17] Soroya S.H., Farooq A., Mahmood K., Isoaho J., Zara S.-E. (2021). From Information Seeking to Information Avoidance: Understanding the Health Information Behavior during a Global Health Crisis. Inf. Process. Manag..

[18] Statista What Sources do you Actively use to Keep Informed about the COVID-19/Coronavirus Pandemic?. https://www.statista.com/statistics/1108009/sources-of-information-about-the-covid-19-corona-pandemic/.

[19] Henrich N., Holmes B. (2010). Communicating During a Pandemic. Health Promot. Pract..

[20] Walter D., Bohmer M., Reiter S., Krause G., Wichmann O. (2012). Risk Perception and Information-Seeking Behaviour during the 2009/10 Influenza A(H1N1)pdm09 Pandemic in Germany. Eurosurveillance.

[21] Wong L.P., Sam I.-C. (2010). Public Sources of Information and Information Needs for Pandemic Influenza A(H1N1). J. Community Health.

[22] Boberg S., Quandt T., Schatto-Eckrodt T., Frischlich L. (2020). Pandemic Populism: Facebook Pages of Alternative News Media and the Corona Crisis-A Computational Content Analysis. https://arxiv.org/pdf/2004.02566.,.

[23] Chao M., Xue D., Liu T., Yang H., Hall B.J. (2020). Media Use and Acute Psychological Outcomes during COVID-19 Outbreak in China. J. Anxiety Disord..

[24] Chen W., Stoecker C. (2020). Mass Media Coverage and Influenza Vaccine Uptake. Vaccine.

[25] Ma K., Schaffner W., Colmenares C., Howser J., Jones J., Poehling K. (2006). Influenza Vaccinations of Young Children Increased With Media Coverage in 2003. Pediatrics.

[26] Shropshire A.M., Brent-Hotchkiss R., Andrews U.K. (2013). Mass Media Campaign Impacts Influenza Vaccine Obtainment of University Students. J. Am. Coll. Health.

[27] Fridman I., Lucas N., Henke D., Zigler C.K. (2020). Association between Public Knowledge About COVID-19, Trust in Information Sources, and Adherence to Social Distancing: Cross-Sectional Survey. JMIR Public Health Surveill..

[28] Bults M., Beaujean D.J., Richardus J.H., Voeten H.A. (2015). Perceptions and Behavioral Responses of the General Public During the 2009 Influenza A (H1N1) Pandemic: A Systematic Review. Disaster Med. Public Health Prep..

[29] Cairns G., De Andrade M., Macdonald L. (2013). Reputation, Relationships, Risk Communication, and the Role of Trust in the Prevention and Control of Communicable Disease: A Review. J. Health Commun..

[30] Hesse B.W., Nelson D.E., Kreps G.L., Croyle R.T., Arora N.K., Rimer B.K., Viswanath K. (2005). Trust and Sources of Health Information. Arch. Intern. Med..

[31] Hesse B.W., Moser R.P., Rutten L.J. (2010). Surveys of Physicians and Electronic Health Information. N. Engl. J. Med..

[32] Jackson D.N., Peterson E.B., Blake K.D., Coa K., Chou W.-Y.S. (2019). Americans&Rsquo; Trust in Health Information Sources: Trends and Sociodemographic Predictors. Am. J. Health Promot..

[33] Marrie R.A., Salter A.R., Tyry T., Fox R.J., Cutter G.R. (2013). Preferred Sources of Health Information in Persons With Multiple Sclerosis: Degree of Trust and Information Sought. J. Med. Internet Res..

[34] Smith D. (2011). Health Care Consumer’s Use and trust of Health Information Sources. J. Commun. Health.

[35] Oberg E.B., Frank E. (2009). Physicians’ Health Practices Strongly Influence Patient Health Practices. J. R. Coll. Physicians Edinb..

[36] van Eimeren B., Ridder C.-M. (2011). Trends in der Nutzung und Bewertung der Medien 1970 bis 2010. Media Perspektiven No. 1. https://www.ard-werbung.de/fileadmin/user_upload/media-perspektiven/pdf/2011/01-2011_Eimeren_Ridder.pdf.

[37] van Eimeren B., Ridder C.-M. (2005). Trends in der Nutzung und Bewertung der Medien 1970 bis 2005. Media Perspektiven No. 10. https://www.ard-zdf-massenkommunikation.de/files/Download-Archiv/MK_2005/10-2005_Eimeren.pdf.

[38] Breunig C., van Eimeren B. (2015). 50 Jahre “Massenkommunikation”: Trends in der Nutzung und Bewertung der Medien No. 11. https://www.ard-werbung.de/media-perspektiven/fachzeitschrift/2015/artikel/50-jahre-massenkommunikation-trends-in-der-nutzung-und-bewertung-der-medien/.

[39] Beisch N., Schäfer C. (2020). Internetnutzung mit großer Dynamik: Medien, Kommunikation, Social Media. Ergebnisse der ARD/ZDF-Onlinestudie 2020. Media Perspektive. https://www.ard-werbung.de/fileadmin/user_upload/media-perspektiven/pdf/2020/0920_Beisch_Schaefer_2020-11-1.pdf.

